# Calcium Signals from the Vacuole

**DOI:** 10.3390/plants2040589

**Published:** 2013-10-14

**Authors:** Gerald Schönknecht

**Affiliations:** Department of Botany, Oklahoma State University, Stillwater, OK 74078, USA; E-Mail: gerald.schoenknecht@okstate.edu; Tel.: +1-405-744-5549; Fax: +1-405-744-7074

**Keywords:** vacuole, ligand-gated Ca^2+^ channel, SV channel, cyclic nucleotide-gated channel, Ca^2+^:H+ exchanger, P-type Ca^2+^-ATPase

## Abstract

The vacuole is by far the largest intracellular Ca^2+^ store in most plant cells. Here, the current knowledge about the molecular mechanisms of vacuolar Ca^2+^ release and Ca^2+^ uptake is summarized, and how different vacuolar Ca^2+^ channels and Ca^2+^ pumps may contribute to Ca^2+^ signaling in plant cells is discussed. To provide a phylogenetic perspective, the distribution of potential vacuolar Ca^2+^ transporters is compared for different clades of photosynthetic eukaryotes. There are several candidates for vacuolar Ca^2+^ channels that could elicit cytosolic [Ca^2+^] transients. Typical second messengers, such as InsP_3_ and cADPR, seem to trigger vacuolar Ca^2+^ release, but the molecular mechanism of this Ca^2+^ release still awaits elucidation. Some vacuolar Ca^2+^ channels have been identified on a molecular level, the voltage-dependent SV/TPC1 channel, and recently two cyclic-nucleotide-gated cation channels. However, their function in Ca^2+^ signaling still has to be demonstrated. Ca^2+^ pumps in addition to establishing long-term Ca^2+^ homeostasis can shape cytosolic [Ca^2+^] transients by limiting their amplitude and duration, and may thus affect Ca^2+^ signaling.

## 1. The Vacuole—A Huge Intracellular Ca^2+^ Store

The large central vacuole of a typical mature plant cell is by far the largest intracellular Ca^2+^ store. It is reasonable to assume that this huge Ca^2+^ store contributes to changes in cytosolic free Ca^2+^ concentrations, [Ca^2+^]_cyt_, during Ca^2+^-mediated intracellular signaling. Usually, the volume of the large central vacuole is about an order of magnitude larger compared to the volume of the cytosol, and the free Ca^2+^ concentration is about three orders of magnitude higher inside the vacuole, compared to the cytosol. The cytosolic free Ca^2+^ concentration at rest, as recorded with ion-selective microelectrodes or fluorescent dyes, is around 200 nM, and may increase to low micromolar concentrations during transient [Ca^2+^]_cyt_ increase [1,2,3,4]. The few published values for vacuolar free Ca^2+^ concentrations, based on measurements with ion-selective microelectrodes, range from 2.3 and 1.5 mM for rhizoids of the liverwort *Riccia fluitans* and root corpus of corn (*Zea mays*), respectively [1], to 200 μM for red beet (*Beta vulgaris*) taproots [5], and the green alga *Eremosphaera viridis* [2]. Total vacuolar Ca^2+^ concentrations can be much higher, more than 60 mM in mesophyll cells of Eudicots [6]. Very little is known about the dynamics of vacuolar free Ca^2+^ concentrations, but the observation that the vacuolar SV channel (see below) is regulated by physiological concentrations of vacuolar Ca^2+^ [7,8] indicates that vacuolar free Ca^2+^ concentrations may change and that these changes probably have physiological effects.

**Figure 1:**
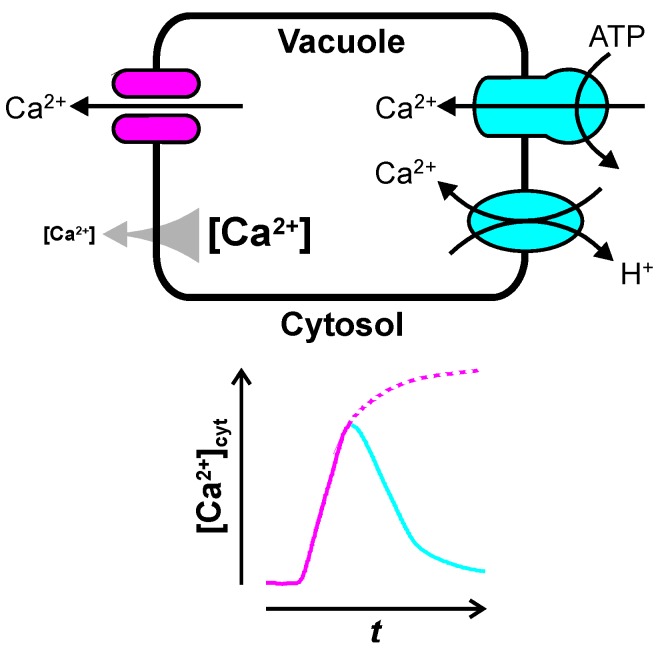
How the vacuole may shape transient changes in cytosolic free Ca^2+^ concentration, [Ca^2+^]_cyt_. A large (≈100 mV) electrochemical potential gradient (gray arrow) pushes Ca^2+^ from the vacuole into the cytosol. As a result, transient opening of vacuolar Ca^2+^ channels (in magenta) elevates [Ca^2+^]_cyt_ (magenta line). Ca^2+^ is pumped back into the vacuole by Ca^2+^-ATPases and Ca^2+^:H^+^ exchangers (in cyan), resulting in a decrease of [Ca^2+^]_cyt_ back to resting levels (cyan line).

About a thousand-fold gradient in free Ca^2+^ concentration plus an electric potential difference across the vacuolar membrane in the range of 0 to −30 mV [2,9] (negative on the cytosolic side [10]) add up to an electrochemical driving force of roughly −100 mV, pushing Ca^2+^ from the vacuole towards the cytosol. As a result, Ca^2+^ release from the vacuole into the cytosol is passive and can be mediated by ion channels, while Ca^2+^ uptake from the cytosol into the vacuole requires energy input and is performed by either ATPases or H^+^-antiporters (Figure 1). The latter use the existing pH gradient of about two pH units [2,11] across the vacuolar membrane for secondary active transport. The opening of vacuolar Ca^2+^ channels increases [Ca^2+^]_cyt_, making these channels good candidates to start or amplify a cytosolic Ca^2+^ signal. This review will summarize our current knowledge of vacuolar Ca^2+^ channels and their involvement in Ca^2+^ signaling. Vacuolar Ca^2+^-ATPases and Ca^2+^:H^+^ exchangers maintain the Ca^2+^ concentration gradient between cytosol and vacuole, and can contribute to restoring low [Ca^2+^]_cyt_. The last part of this review will summarize recently emerging knowledge about how vacuolar Ca^2+^ pumps may shape cytosolic [Ca^2+^] transients. For further information the reader is referred to a number of excellent reviews that have been published recently, summarizing vacuolar ion transport [12,13,14], plant Ca^2+^ transporter [15,16,17,18,19], organellar Ca^2+^ transport [20], or vacuolar Ca^2+^ transport [21,22].

## 2. Ca^2+^ Release from the Vacuole

Vacuolar Ca^2+^ release seems a well-established step of intracellular signal transduction in plant cells. In plant physiology textbooks for example, ABA-induced stomatal closure is usually depicted as being mediated by the opening of vacuolar Ca^2+^ channels. However, there is a lack of solid evidence for a physiological stimulus triggering release of Ca^2+^ from the large central vacuole of an intact cell. ABA-induced increase in guard cell [Ca^2+^]_cyt_, visualized by digital ratio imaging of fluorescent Ca^2+^ indicators, has been reported to be more pronounced close to the vacuole [23,24,25]. Similar reports exist for transient increases in guard cell [Ca^2+^]_cyt_ triggered by high external Ca^2+^ concentrations [26] or by plasma membrane hyperpolarization [27]. Yet as some of these reports mention, better spatial and temporal resolution is required before definitive statements about the contributing Ca^2+^ stores can be made. Even though the experimental techniques for intracellular Ca^2+^ imaging have improved in recent years, no progress has been reported in imaging vacuolar Ca^2+^ release.

Another approach to detect vacuolar Ca^2+^ release was to record luminescence from transgenic *Arabidopsis thaliana* seedlings expressing the Ca^2+^-sensitive photoprotein aequorin either in the cytosol or anchored to the cytosolic side of the vacuolar membrane. [Ca^2+^]_cyt_ spikes were induced by cooling [28] or addition of mannitol [29], and slightly different kinetics in luminescence of cytosolic *versus* vacuole-bound aequorin were interpreted as indication for vacuolar Ca^2+^ release. While these recordings are compatible with vacuolar Ca^2+^ release, alternative explanations cannot be ruled out. Only 24% of aequorin activity could be detected on isolated vacuoles [28], raising the possibility that other subcellular domains than the “vacuolar microdomain” contributed to recorded signals. Ca^2+^-dependent luminescence was recorded from intact *A*. *thaliana* seedlings, six to seven days old, and different cell types may have contributed to recorded signals to a varying degree in different transgenic lines. Repeating the pioneering work with aequorin using other recombinant Ca^2+^ indicators could provide important new insights into vacuolar Ca^2+^ release.

### 2.1. Inositol Trisphosphate-Dependent Vacuolar Ca^2+^ Release

**Figure 2:**
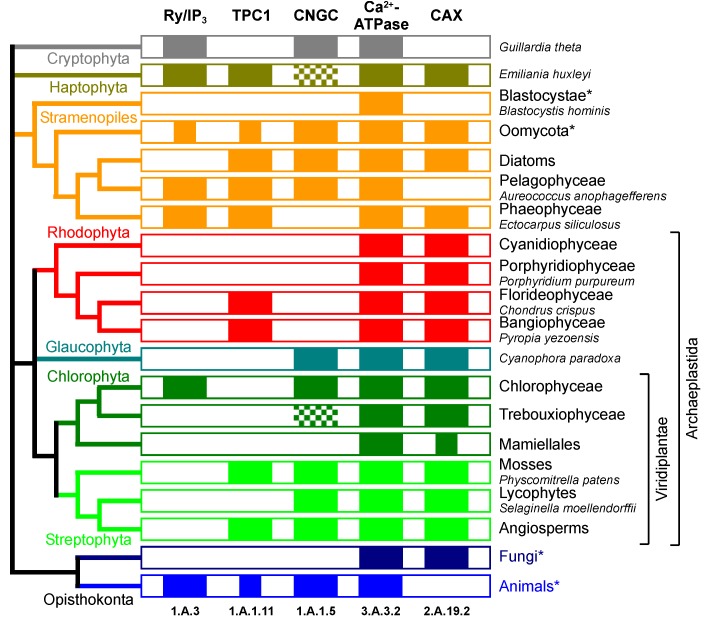
Phylogenetic distribution of Ca^2+^ transporters in photosynthetic eukaryotes. Colored blocks indicate the presence of a certain Ca^2+^ transport protein (top and bottom labels) in a specific clade (labels on left and right). Abbreviations used for Ca^2+^ transport proteins: Ry/IP_3_, ryanodine-InsP_3_ receptor Ca^2+^ channel (TC 1.A.3); TPC1, two-pore Ca^2+^ channel (TC 1.A.1.11.13/18/19/22); CNGC, cyclic nucleotide-gated cation channel (TC 1.A.1.5); Ca^2+^-ATPase (TC 3.A.3.2); CAX, Ca^2+^:H^+^ exchanger (TC 2.A.19.2). Smaller block indicates that not all species in this clade seem to contain the Ca^2+^ transporter (see text). Checkered blocks indicate that it is currently not clear whether Haptophyta and Trebouxiphyceae contain cyclic nucleotide-gated cation channels or a similar cation channel from a different family of the voltage-gated ion channel superfamily (TC 1.A.1). The phylogenetic relationship (left [39]) of major eukaryotic photosynthetic clades (listed right) is presented and color coded (according to labels on the left). Clades marked with an asterisk are non-photosynthetic. For clades containing only one species with a fully sequenced genome, the species is given. Ca^2+^ transporters were identified by comparing established members from each Ca^2+^ transporter family to proteoms from selected eukaryotes with completely sequenced genomes using BLAST, followed by verification of each candidate by BLAST at TCDB [40,41].

In animal cells D-*myo*-inositol 1,4,5-trisphosphate (InsP_3_) binds to a family of Ca^2+^ channels, the inositol trisphosphate receptor, located in the ER, resulting in Ca^2+^ release into the cytosol [30,31]. In plant cells different stimuli elicit an increase in InsP_3_, and InsP_3_ has been shown to cause various physiological responses [32,33]. These physiological responses to InsP_3_ are probably mediated by [Ca^2+^]_cyt_. Increase of cytosolic InsP_3_ by release from a caged photoactivatable derivative [34,35] or by microinjection [36] result in a transient increase in [Ca^2+^]_cyt_. However, in plant cells the molecular mechanisms of InsP_3_-induced [Ca^2+^]_cyt_ increase are not well understood, since the typical InsP_3_ receptor Ca^2+^ channel found in animals seems to be missing in land plants (Embryophyta). None of the more than 50 sequenced land plant genomes seems to encode an ortholog of the InsP_3_ receptor (Figure 2). To reconcile *in vivo* evidence for InsP_3_-mediated signal transduction in plant cells, with the seeming lack of an InsP_3_ receptor homolog two explanations have been offered. First, the similarity between land plant and animal InsP_3_ receptor Ca^2+^ channels might be too low to be detected due to early evolutionary divergence [37]. Alternatively, there might be no InsP_3_ receptor ortholog in land plants, and an InsP_3_-dependent Ca^2+^ release mechanism might have evolved from different membrane transport proteins [38].

Surprisingly, an InsP_3_ receptor was detected as a very abundant component of the flagellar proteome of *Chlamydomonas reinhardtii* [42]. The genomes of both Chlorophyceae sequenced so far, namely *C*. *reinhardtii* [43] and *Volvox carteri* [44], encode a single InsP_3_ receptor homolog. Pharmacological studies indicate that InsP_3_ is involved in Ca^2+^-dependent deflagellation observed in *C*. *reinhardtii* upon application of pH, osmotic or temperature shock [33,45]. The existence of InsP_3_ receptors in Chlorophyceae gave rise to speculation that this Ca^2+^ channel has been present in the common ancestor of green algae and land plants, and was lost in land plants [46]. However, InsP_3_ receptor homologs are not detected in other classes of green alga, such as Prasinophyceae (Mamiellales) or Trebouxiophyceae (Figure 2). For *E*. *viridis*, a Trebouxiophyceae, injection of InsP_3_ has been shown to trigger the opening of Ca^2+^-dependent plasma membrane K^+^ channels, which together with pharmacological evidence indicates the existence of an InsP_3_-dependent Ca^2+^ release mechanism in this green alga [47]. Homologs of animal InsP_3_ receptor Ca^2+^ channels also do not seem to be present in the glaucophyte *Cyanophora paradoxa* [48] or in any of the five red algae (Rhodophyta) with sequenced genomes. Currently, it seems as if Chlorophyceae are the only class within Archaeplastida (red algae & green plants & glaucophytes) containing InsP_3_ receptor homologs (Figure 2). While this patchy distribution of InsP_3_ receptors in Archaeplastida could be the result of multiple losses in different lineages, acquisition via horizontal gene transfer [49], seems more likely. In embryos of the brown alga (Phaeophyceae) *Fucus serratus*, photolysis of caged InsP_3_ caused an increase in [Ca^2+^]_cyt_ [50]. The genome of the brown alga *Ectocarpus siliculosus* [51] does seem to encode an InsP_3_ receptor homolog, as do some other Stramenopile genomes (Figure 2).

Most attempts to identify the nature of the InsP_3_-sensitive Ca^2+^ store in plant cells produced results that point to the vacuole [32]. InsP_3_-dependent Ca^2+^ release was observed with tonoplast-enriched vesicles from oat (*Avena sativa*) roots [52] and from beet taproots [53], and with isolated intact vacuoles from sycamore (*Acer pseudoplatanus*) [54] and *Chenopodium album* [55] suspension culture cells. Yet some binding studies with [3H]InsP3 [56], or with antibodies raised against human InsP_3_ receptor Ca^2+^ channels [57] indicate that InsP_3_ binds to other membranes than the tonoplast.

Two groups in the early 1990s reported patch-clamp recordings of InsP_3_-dependent ion currents with intact isolated vacuoles from red beet taproots [58,59,60]. Whole vacuole currents induced by application of 1 μM InsP_3_ were comparable (when applying the same sign convention [10]), and estimated (single channel current) reversal potentials came close to the Nernst potential of Ca^2+^ indicating Ca^2+^-selectivity. Yet single channel characteristics greatly differed. Single channel I/V curves were linear in one publication [58] while they increased non-linearly at more negative voltages in the other [60]. Moreover, neither channel gating nor open channel conductance levels, reported by the two groups did match [58,59,60]. Surprisingly, InsP_3_-induced whole vacuole currents did not seem to be affected by the Ca^2+^ concentration on the cytosolic side [60]. This is different from animal InsP_3_ receptors, which are activated by cytosolic Ca^2+^ [30,31]. Attempts by several other groups to record InsP_3_-dependent vacuolar ion currents were unsuccessful [61,62,63]. With Ca^2+^-selective microelectrodes, no InsP_3_-induced Ca^2+^ release was detected with isolated intact vacuoles from beet taproots [64]. It has been suggested that a hyperosmolar incubation of red beet tissue before vacuole isolation is essential to activate InsP_3_-dependent vacuolar ion channels [60]. This seems to be in line with observations that a decrease of bath osmolarity increases InsP_3_-induced current density in patch-clamp recordings [60] as well as vacuolar Ca^2+^ efflux rates recorded with the fluorescent indicator Quin 2 [54]. Moreover, for vacuoles isolated from sycamore suspension culture cells a clear dependence of InsP_3_-dependent Ca^2+^ release on culture age was observed, increasing in parallel to cell fresh weight over time [54]. It therefore seems possible that InsP_3_-depedent vacuolar Ca^2+^ release may only be detected with cells at a certain developmental stage and when a slight hypoosmolar stress is applied to isolated vacuoles. Since the pioneering work on InsP_3_-dependent vacuolar Ca^2+^ release in the late 1980s and early 1990s little new experimental work on this important topic has been reported. The answer to the question if there is InsP_3_-dependent vacuolar Ca^2+^ release, is still that there are several evidences for it, but the molecular mechanisms are unclear.

### 2.2. Ca^2+^ Mobilization by Inositol Hexakisphosphate

Studies with guard cells from potato (*Solanum tuberosum*) and broad bean (*Vicia faba*) indicated that ABA-induced [Ca^2+^]_cyt_ increase and stomatal closure might be mediated by *myo*-inositol hexakisphosphate (InsP_6_, a.k.a. phytate) [65]. Corroborating these observations, photoactivation of caged InsP_6_ in broad bean guard cells resulted in a transient increase in [Ca^2+^]_cyt_ [66]. Application of 5 μM InsP_6_ to isolated broad bean guard cell vacuoles increased whole-vacuole current amplitudes [66]. These InsP_6_-dependent vacuolar currents are different from vacuolar currents induced by 1 μM InsP_3_ [58,59,60]. Even when recorded with a 10^6^-fold [Ca^2+^] gradient (10 mM in the vacuole and ≈2 nM outside), InsP_6_-dependent vacuolar currents did not show strong rectification, indicating that ions other than Ca^2+^ were conducted, and current reversal potentials were close to 0 mV, indicating a lack of Ca^2+^-selectivity. Therefore, there seems to be no electrophysiological evidence for Ca^2+^ mobilization by InsP_6_ from the large central vacuole.

### 2.3. Cyclic Adenosine Diphosphoribose (cADPR)-Induced Vacuolar Ca^2+^ Release

Cyclic adenosine diphosphoribose (cADPR) is generated from NAD^+^ by ADP-ribosyl cyclases and functions as a Ca^2+^ mobilizing second messenger in different eukaryotic clades [67]. In animal cells, cADPR controls Ca^2+^ release from the ER by activating ryanodine receptor Ca^2+^ channels [68,69]. While InsP_3_ receptors (see above) are activated by direct binding of InsP_3_, the mechanism(s) by which cADPR activates ryanodine receptors is unclear [70,71]. Isolated, intact ryanodine receptor Ca^2+^ channels reconstituted into planar lipid bilayers are not affected by cADPR, indicating that cADPR does not directly bind to ryanodine receptors [72,73,74]. Instead, cADPR might bind to a secondary protein, which regulates ryanodine receptor activity [71,75], or cADPR might elevate luminal [Ca^2+^], which is known to activate ryanodine receptors [74].

Even though no homolog of ryanodine receptor Ca^2+^ channels can be detected in land plants (Figure 2), there are evidence that cADPR does function as a Ca^2+^ mobilizing second messenger in plant cells—a situation comparable to InsP_3_. Early work from Dale Sanders’ group demonstrated cADPR-dependent Ca^2+^ release from vacuolar-enriched microsomes of red beet taproots [53,76]. Later work indicated that systemin-induced elevation of [Ca^2+^]_cyt_ observed after wounding tomato (*Lycopersicon esculentum*) leaves was in part caused by cADPR-dependent Ca^2+^ release from internal stores [77]. In *A*. *thaliana* circadian [Ca^2+^]_cyt_ oscillations were reported to be the result of circadian oscillations of cytosolic cADPR concentrations [78]. Yet this is a matter of debate [79,80], and a regulation of circadian [Ca^2+^]_c__yt_ oscillations in *A*. *thaliana* by InsP_3_ has been reported as well [81]. Intracellular Ca^2+^ release by cADPR seems to be an essential step of ABA signal transduction [82,83]. ABA activates ADP-ribosyl cyclases and elevates cADPR concentrations triggering the expression of ABA-responsive genes [82,84]. During ABA-induced stomatal closure, cADPR causes an increase in [Ca^2+^]_cyt_ resulting in potassium salt release and guard cell turgor loss [83,85].

In the unicellular green alga *E*. *viridis*, pharmacological experiments with ryanodine, ruthenium red, and different caffeine analogs indicated a ryanodine receptor-like Ca^2+^ release mechanism [86,87]. However, no ryanodine receptor homolog can be detected in sequenced genomes of two Trebouxiophyceae (Figure 2), the class of green alga to which *E*. *viridis* belongs. It should be mentioned that the Ca^2+^ channels in Chlorophyceae and brown algae (Phaeophyceae), mentioned above as possible InsP_3_ receptors, belong to the same ion channel family (TC 1.A.3) as ryanodine receptors. Due to low sequence conservation, phylogenetic studies do currently not allow a clear assignment of these algal Ca^2+^ channels to either InsP_3_ or ryanodine receptor subfamily [88].

Early studies provided evidence that cADPR acts on Ca^2+^ channels in the vacuolar membrane [53,76]. Later studies from the same group indicated that in addition to the vacuole, the ER might also function as cADPR-sensitive Ca^2+^ store in plant cells [89]. Patch-clamp recordings with isolated vacuoles from red beet tap roots [53] or from broad bean guard cells [83] detected currents elicited by cADPR. Current reversal potentials indicated Ca^2+^ selectivity of cADPR-dependent whole vacuole currents [83]. As mentioned above, isolated ryanodine receptor Ca^2+^ channels from animals are not gated open by cADPR [72,73,74]. This seems to be in contrast to the cADPR-dependent currents recorded from plant vacuoles. Moreover, cADPR-dependent vacuolar currents were largest at low [Ca^2+^]_cyt_ (≈10 nM) and decreased at increasing [Ca^2+^]_cyt_, almost disappearing at 1 μM [83]. In contrast, animal ryanodine receptors are involved in Ca^2+^-induced Ca^2+^ release, showing increasing open probabilities at increasing [Ca^2+^]_cyt_ [69,70]. The inhibition of cADPR-dependent vacuolar currents by micromolar [Ca^2+^]_cyt_ might explain why recordings with Ca^2+^-selective microelectrodes in media containing [Ca^2+^] ≈ 2 μM failed to detect a cADPR-dependent Ca^2+^ efflux from isolated vacuoles [64]. A more detailed characterization of cADPR-elicited vacuolar currents is urgently needed. Results so far suggest that cADPR-dependent vacuolar Ca^2+^ release is regulated in a different way than animal ryanodine receptors are.

### 2.4. The Slow Vacuolar / Two Pore Channel (SV/TPC1)

The first patch-clamp studies with isolated plant vacuoles resulted in the description of a voltage-dependent, slowly activating, Ca^2+^ regulated, non-selective cation channel—the slow vacuolar (SV) channel [90,91]. It soon became clear that this channel is present in all land plants (Embryophyta) and in each cell type containing a large central vacuole [92,93]. Almost 20 years after these initial patch-clamp studies, the SV channel was shown to be encoded by the *TPC1* (two pore channel) gene [94], which is present in one or two copies in all Embryophyta. This finally allowed the application of genetic manipulations to study vacuolar Ca^2+^ release. Only the aspect of vacuolar Ca^2+^ release will be covered here. For other possible physiological functions of the SV/TPC1 channel the reader is referred to a recent comprehensive review [95].

#### 2.4.1. Is There Ca^2+^-Induced Ca^2+^ Release by the SV Channel?

It took several years before it was realized that the SV channel is Ca^2+^ permeable and might thus function as vacuolar Ca^2+^ channel [96]. Earlier reports of voltage-dependent vacuolar Ca^2+^ channels [62,97] probably were the result of SV channel recordings, and it was proposed [98] that the voltage-gated Ca^2+^ channel described in vacuoles from sugar beet (*Beta vulgaris*) tap roots [99,100] does reflect SV channel activity as well. Detailed biophysical studies confirmed a selectivity of SV channels for divalent cations [101,102]. Yet SV channels show a strong rectification allowing cation uptake into the vacuole but allowing little, if any, cation release from the vacuole. In other words, SV currents are usually recorded at positive membrane potentials, while the vacuole has a slightly negative potential [2,9]. To allow vacuolar Ca^2+^ release, some factor has to open the SV channel at physiological potentials (or the membrane potential has to change).

When Ward and Schroeder [96] discovered that the SV channel is permeable for Ca^2+^, they combined this with the fact that increasing [Ca^2+^]_cyt_ increases the open probability and postulated Ca^2+^-induced Ca^2+^ release by the SV channel. In animal cells, an initial small [Ca^2+^]_cyt_ increase is amplified via Ca^2+^-induced Ca^2+^ release by ER ryanodine and InsP_3_ receptor Ca^2+^ channels [31,69]. It was tempting to assume that the SV channel might mediate Ca^2+^-induced Ca^2+^ release in plant cells. However, a detailed investigation on how SV channel open probability is regulated by cytosolic [Ca^2+^], vacuolar [Ca^2+^], and trans-tonoplast electrical potential seemed to exclude Ca^2+^-induced Ca^2+^ release. As long as the electrochemical potential gradient for Ca^2+^, Δμ_Ca_, would drive Ca^2+^ efflux, SV channel open probability was extremely low, and only at unphysiological conditions creating a Δμ_Ca_ driving Ca^2+^ into the vacuole, higher open probabilities were observed [103]. It almost seemed as if the SV channel were regulated by cytosolic [Ca^2+^], vacuolar [Ca^2+^], and membrane potential in a way to prevent vacuolar Ca^2+^ release. In the following years several studies presented evidence for [104,105,106] or against [5,7,107] Ca^2+^-induced Ca^2+^ release by the SV channel. As a result of these studies and others it was realized just how complex the regulation of SV channels is. In addition to Ca^2+^ and membrane potential, reducing agents increase SV channel activity, while oxidizing agents abolish it [108]; cytosolic Mg^2+^ activates SV channels, comparable to Ca^2+^ [105,106], while vacuolar Mg^2+^-comparable to vacuolar Ca^2+^-inhibits SV channel activity [7]; likewise vacuolar Na^+^ inhibits SV channel activity [107].

Measurement of SV channel activity by whole vacuole patch-clamp recording reports net charge movement and does not provide direct information about Ca^2+^ fluxes. Yet direct measurements of Ca^2+^ fluxes with isolated vacuoles resulted in seemingly conflicting results. Using Ca^2+^-selective microelectrodes, an increase in vacuolar Ca^2+^ efflux was recorded upon addition of 5 or 10 μM Ca^2+^ as well as upon addition of ABA, calmodulin, cAMP, or ATP [64]. Yet due to technical limitations, these recordings were performed at [Ca^2+^]_cyt_ ≈ 2 μM, and one could question the physiological relevance of a vacuolar Ca^2+^ release brought about by an elevation of [Ca^2+^]_cyt_ from 2 μM to 5 μM. For comparison, in animals cells the InsP_3_ receptor has maximum activity at [Ca^2+^]_cyt_ ≈ 300 nM [31], mediating Ca^2+^-induced Ca^2+^ release from *sub*micromolar to low micromolar concentrations. In another study, combining patch-clamp with ratiometric fluorescence imaging of Ca^2+^, no SV-mediated Ca^2+^ release from the vacuole could be recorded, while Ca^2+^ flux into the vacuole was recorded [109]. Remarkably, at −10 mV negative currents, *i.e*., net cation (K^+^) efflux from the vacuole was recorded electrophysiologically, while fura-2 fluorescence indicated opposite Ca^2+^ flux-into the vacuole. This is in line with a multi-ion single-file permeation mechanism proposed for the SV channel [110], where different cations get alternating access to the same pore allowing movement of different cations in opposite directions. These elegant recordings with fura-2 loaded into the tip of a patch pipette [109] demonstrate that negative whole vacuole currents cannot be equated with Ca^2+^ efflux from the vacuole.

The author of this review has always been skeptical of Ca^2+^-induced Ca^2+^ release by the SV channel and would argue that vacuolar Ca^2+^ release by the SV channel under physiologically relevant conditions still has to be demonstrated.

#### 2.4.2. Is TPC1 Involved in Ca^2+^ Signaling?

The SV channel consists of a dimer of the TPC1 (two pore channel) protein [94]. Each TPC1 has two pore domains, as indicated by the name, and four pore domains form a functional voltage-dependent ion channel. A single *TPC1* gene is found in most land plants (Embryophyta), others have two genes, likely due to recent gene duplication. In the genome of the lycophyte *Selaginella moellendorffii* [111], no *TPC1* gene has been annotated. TPC1 shows a patchy distribution among photosynthetic eukaryotes (Figure 2) [95]. It seems to be lacking from Chlorophyta [46]. Yet patch-clamp recordings with isolated vacuoles from the green alga *E*. *viridis* resulted in whole vacuole currents reminiscent of SV currents, showing outward rectification, slow activation, Ca^2+^ dependence, inhibition by acidification, and blockage by Zn^2+^ [112]. There are no sequence data available for *E*. *viridis*, but the genomes of two other Trebouxiophyceae (*Coccomyxa subellipsoidea* [113] and *Chlorella variabilis* [114]) do not seem to encode TPC1. Among the five red algal genomes sequenced, only *Chondrus crispus* [115] and *Pyropia yezoensis* [116] seem to contain a *TPC1* gene. As other Ca^2+^ channels, TPC1 has a patchy distribution among Archaeplastida (Figure 2), and it is hard to decide whether the last common ancestor of Archaeplastida had a TPC1 gene, which was lost in several lineages, or whether two lineages of the Archaeplastida acquired a TPC1 gene via horizontal gene transfer [49]. TPC1 channels were originally discovered in rats [117], and are wide spread in vertebrates, while absent in insects and fungi.

In mammalian cells, TPC’s (there are more subfamilies than just TPC1) are localized in acidic organelles, the endolysosomal compartment, where they are activated by NAADP (nicotinic acid adenine dinucleotide phosphate) causing Ca^2+^ release [118,119,120]. NAADP, which is produced from NADP^+^ by ADP-ribosyl cyclases (the same enzymes converting NAD^+^ into cADPR [121]) had been known as a Ca^2+^-mobilizing second messenger for several years [122]. NAADP-elicited Ca^2+^ release by mammalian TPC channels does not behave as a Ca^2+^-induced Ca^2+^ release system [123,124]. Instead, the different Ca^2+^ pools and messengers in mammalian cells are supposed to work together in the following way: NAADP triggers Ca^2+^ release from endolysosomes via TPC channels resulting in a small (and possibly localized) increase in [Ca^2+^]_cyt_; this activates (nearby) InsP_3_ and ryanodine receptors, and Ca^2+^-induced Ca^2+^ release from the ER causes an effective (and widespread) [Ca^2+^]_cyt_ elevation [120,125]. However, the function of mammalian TPC channels as NAADP-dependent Ca^2+^ channels has recently been questioned. It has been reported that TPC proteins are activated by the phosphoinositide PIns(3,5)P_2_ instead, and that they function as Na^+^ channels, mediating Na^+^ efflux to depolarize lysosome membranes [126]. Moreover, it has been questioned whether NAADP binds directly to the TPC channel or to an accessory component [127]. Little is known about the NAADP or PIns(3,5)P_2_ in plants. A study with microsomal vesicles from red beet and cauliflower (*Brassica oleracea*) observed NAADP-dependent Ca^2+^ release from ER vesicles but not from vacuolar vesicles [128]. Still, it might be worthwhile to investigate the effect of NAADP or PIns(3,5)P_2_ on SV/TPC1 channel activity.

With the coding gene identified, mutants of *TPC1* should give insight into the physiological function of SV/TPC1 channels. Surprisingly, an *A*. *thaliana* T-DNA-knockout line, *attpc1-2*, lacking the TPC1 protein does not show obvious phenotypic differences compared to wild-type plants, even though *AtTPC1* is a single copy gene [94]. However, some more ‘subtle’ differences were observed. For *attpc1-2* mutants, ABA was a less effective inhibitor of seed germination, and *TPC1*-overexpresing lines were ABA-hypersensitive. ABA-induced stomatal closure was not affected by the absence or presence of TPC1, yet stomatal closure by high external Ca^2+^ was not observed in *tpc1-2* mutants in contrast to wild-type plants. These results were taken as an indication for vacuolar Ca^2+^ release by the SV/TPC1 channel [94]. However, as shown later, [Ca^2+^]_cyt_ changes induced by high external Ca^2+^ were identical in *attpc1-2* and wild-type plants, indicating a role of AtTPC1 in stomatal closure in a signal pathway downstream of [Ca^2+^]_cyt_ [129,130]. To further elucidate the function of AtTPC1 in stress-induced [Ca^2+^]_cyt_ increase, [Ca^2+^]_cyt_ was recorded in *attpc1-2* knockout, wild-type, and *TPC1*-overexpressing lines during the application of abiotic stresses (cold, hyperosmotic, salt, and oxidative) or biotic factors (elf18, flg22) [130]. For all stresses and biotic factors tested there was no difference in amplitude or time course of recorded [Ca^2+^]_cyt_ transients between lines lacking or overexpressing *AtTPC1*. Moreover, expression of stress-induced genes known to require [Ca^2+^]_cyt_ increase did not show any dependence on TPC1 either. This seems to rule out an involvement of AtTPC1 in [Ca^2+^]_cyt_ increase induced by the different tested stresses and elicitors [130]. It is important to emphasize that some of those stresses (cold, hyperosmotic, and salt) have been reported to cause vacuolar Ca^2+^ release based on recordings with vacuole-bound aequorin (see above) [28,29].

While the loss-of-function mutant *attpc1-2* has a wild-type phenotype, in the gain-of-function *fou2* (fatty acid oxygenation upregulated 2) mutant older leaves have shorter petioles, show slight epinasty, and accumulate more anthocyanins, comparable to other mutants overaccumulating the plant hormone jasmonic acid [131]. In *fou2*, a point mutation (D454N) reduces the sensitivity of SV/TPC1 channels against inhibition by vacuolar Ca^2+^ and H^+^ [132]. This is expected to result in increased channel activity in the mutant. Yet somewhat counter-intuitive, *fou2* mutants compared to wild-type plants accumulate higher vacuolar [Ca^2+^] and lower vacuolar [K^+^] [132], and display a transcript profile reminiscent of a K^+^ starvation transcriptome [133]. This has been explained by loss of K^+^ from vacuoles due to an overactive SV/TPC1 channel in the *fou2* mutant, being compensated by Ca^2+^ uptake into the vacuole by the Ca^2+^:H^+^ antiporter CAX3 [132], which is upregulated under K^+^ deficiency [134]. However, comparing different cell types within *A*. *thaliana* leaves, epidermal cells with a higher *AtTPC1* transcript level, compared to mesophyll cells, have lower vacuolar [Ca^2+^], which increases in *Attpc1-2* mutants [135]. SV/TPC1 channels seem to influence vacuolar [Ca^2+^]. However, whether this indicates that the SV/TPC1 channel functions as a vacuolar K^+^ efflux channel [95,132], or as a Ca^2+^ efflux channel [135,136], or as a vacuolar Ca^2+^ sensor [8,136] seems currently not possible to differentiate.

While studies mentioned so far seem to provide little support for a function of TPC1 in Ca^2+^ signaling, there are a few observations that still might support such a function. Application of high concentrations of sucrose induce a transient increase in [Ca^2+^]_cyt_ in plant cells [137]. This sucrose-induced [Ca^2+^]_cyt_ increase was enhanced in plants overexpressing *TPC1* and reduced in plant lines with suppressed *TPC1* expression [138,139]. In tobacco (*Nicotiana tabacum*) BY-2 cells, a transient increase in [Ca^2+^]_cyt_ induced by the fungal elicitor cryptogein was reduced in cell lines with suppressed *NtTPC1* expression, as were defense-related gene expression and programmed cell death [139]. Similar, in rice (*Oryza sativa*) a transient increase in [Ca^2+^]_cyt_ induced by the fungal elicitor TvX was reduced in *Ostpc1* knockout mutants [140]; elicitor-induced defense responses were reduced in *Ostpc1* mutants and enhanced in *OsTPC1* overexpressing lines [141]. In this context it has to be mentioned that the intracellular localization of TPC1 in monocots, and especially in rice, has been debated until recently. In *A*. *thaliana* and other dicots the vacuolar localization of TPC1 is well established [94,95,142], and recordings of typical SV channel currents from vacuoles of different monocots [93,143,144,145] strongly suggest a vacuolar localization as well. However, in rice, anti-OsTPC1 antibodies seem to bind to the plasma membrane of intact protoplasts and to plasma membrane-enriched subcellular fractions from suspension-cultured rice cells [140,146]. Patch-clamp recording of characteristic SV channel activity from vacuolar membranes of wild-type rice, but not from *Ostpc1* knock-out lines recently clarified that rice OsTPC1 forms the vacuolar SV channel—as in other land plants [147].

It is currently not understood why the large central vacuole of plant cells contains thousands of SV/TPC1 channels. Would all these cation channels open, the resulting huge fluxes would probably collapse existing cation gradients within 10 to 20 s, with disastrous consequences for the cell. But then, these channels seem to be closed (almost?) all the time, and the suggestion that “*Arabidopsis* is better off without” TPC1 [95] seems to hold for plants growing in a green house. In a computational model for the guard cell based on existing knowledge about transport proteins, it was observed that the SV/TPC1 channel “is unsuited to the task of Ca^2+^ release using any of the range of gating and permeation parameters that are reported in the literature” [148]. To describe vacuolar Ca^2+^ release in this guard cell computational model, a Ca^2+^ channel similar to animal InsP_3_ or ryanodine receptors was included, with properties so far not observed for vacuolar Ca^2+^ channels [149].

### 2.5. Cyclic Nucleotide-Gated Channels

Cyclic nucleotide-gated channels AtCNGC19 and AtCNGC20 were recently reported to be targeted to the vacuolar membrane [150]. However, another group at the same time reported AtCNGC20 to be targeted to the plasma membrane [151]. CNGCs are nonselective cation channels, some conducting Ca^2+^, regulated by cAMP, cGMP, and calmodulin [152,153,154]. While nothing is currently known about the ion selectivity of AtCNGC19 and AtCNGC20, they might potentially be involved in vacuolar Ca^2+^ release. These two CNGC’s from *A*. *thaliana* seem to be involved in ion homeostasis and nutrient signaling. Expression of *AtCNGC19* and *AtCNGC20* is upregulated within hours after applying salt stress (200 mM NaCl) [155]. Expression of *AtCNGC19* is upregulated within hours after applying boron deficiency (transfer from 2 μM to 0 μM B) in parallel to an increase in [Ca^2+^]_cyt_ [156]. Cyclic nucleotide-gated cation channels seem to be present in most photosynthetic eukaryotes with the exception of red algae (Rhodophyta) (Figure 2). Yet most of these cyclic nucleotide-gated channels are probably not located in the vacuole but in other membranes—as in *A*. *thaliana*.

### 2.6. Transient Receptor Potential Ca^2+^ Channels

In fungi, Ca^2+^ is released from the large central vacuole by a Ca^2+^ channel belonging to the transient receptor potential (TRP) family (Yvc1 in *Saccharomyces cerevisiae*; TC 1.A.4.4.1) [157,158]. No genes encoding orthologs of TRP Ca^2+^ channels have been detected in genomes of higher plants, while some green algae may contain TRP Ca^2+^ channels [46]. Yet no TRP Ca^2+^ channel outside Opisthokonta has been characterized so far, which is why this Ca^2+^ channel family is not included in Figure 2.

## 3. Ca^2+^ Uptake into the Vacuole

Ca^2+^ uptake into the large central vacuole is an essential part of cytosolic Ca^2+^ homeostasis and—in contrast to Ca^2+^ release—the molecular mechanisms of vacuolar Ca^2+^ uptake are well understood. P-type Ca^2+^-ATPases and Ca^2+^:H^+^ exchanger move Ca^2+^ against its electrochemical potential gradient from the cytosol into the vacuole (Figure 1). These Ca^2+^ pumps establish and maintain the electrochemical potential gradient driving Ca^2+^ release via vacuolar Ca^2+^ channels (see above). Most work on vacuolar Ca^2+^ pumps has concentrated on the aspect of ion homeostasis, which will not be covered here. In addition to establishing a Ca^2+^ gradient, vacuolar Ca^2+^ pumps are likely to contribute to shaping [Ca^2+^]_cyt_ transients in the cytosol. High activity of Ca^2+^ pumps, e.g., is expected to decrease amplitude and duration of [Ca^2+^]_cyt_ transients (Figure 1). Especially in signal transduction pathways where [Ca^2+^]_cyt_ transients do not just function as a simple switch [159], but where the shape of the [Ca^2+^]_cyt_ transients is important [160], the activity of vacuolar Ca^2+^ pumps is likely to play a role. Different stimuli have been shown to result in [Ca^2+^]_cyt_ transients of different shape and duration in plants cells [28,160,161], and the same stimulus, such as salt stress (NaCl), can cause [Ca^2+^]_cyt_ transients that increase in amplitude with stimulus intensity [162]. Attempts to describe [Ca^2+^]_cyt_ oscillations by mathematical models nicely show that amplitude and frequency depend on both, Ca^2+^ influx into the cytosol and Ca^2+^ removal from the cytosol by Ca^2+^ pumps [86]. In guard cells it has been shown that [Ca^2+^]_cyt_ oscillations with different frequencies have different effects on stomatal closure [163]. How vacuolar Ca^2+^ pumps may affect intracellular signal transduction by shaping [Ca^2+^]_cyt_ transients (Figure 1) is slowly beginning to emerge [15,17].

### 3.1. P-Type Ca^2+^-ATPases

P-type Ca^2+^-ATPases function as high affinity low turnover Ca^2+^ pumps, operating as ATP-driven Ca^2+^:H^+^ exchanger. Flowering plants (angiosperms) seem to contain more than a dozen Ca^2+^-ATPase genes, and plasma membrane and each organelle seem to contain at least one Ca^2+^-ATPase [164,165]. Ca^2+^-ATPases, as P-type ATPases in general, are phylogenetically very old and have been detected in all eukaryotic clades [166] (Figure 2). In *A*. *thaliana*, two Ca^2+^-ATPases are targeted to the vacuolar membrane, with ACA4-GFP detected in small vacuoles of transiently transformed protoplasts [167] and ACA11-GFP detected in the large central vacuole of root cells [168]. Both vacuolar Ca^2+^-ATPases contain an *N*-terminal autoinhibitory domain with a calmodulin binding site.

Knockout mutants, lacking either ACA4 or ACA11, did not show obvious phenotypic differences compared to wild-type *A*. *thaliana* plants, while double knockout lines developed hypersensitive-like lesions [169]. Total leaf Ca^2+^ was not changed by the double knockout, and lesions could be suppressed by blocking salicylic acid production or accumulation, indicating that the lack of vacuolar Ca^2+^-ATPases resulted in hyperactivity of the salicylic acid-dependent programmed cell death pathway. It was suggested that vacuolar Ca^2+^-ATPases shape [Ca^2+^]_cyt_ transients that trigger programmed cell death, but no [Ca^2+^]_cyt_ transients were recorded [169]. The first direct evidence that lack of a vacuolar Ca^2+^-ATPase can change the shape of a [Ca^2+^]_cyt_ transient came from the moss *Physcomitrella patens*. When the Ca^2+^-ATPase PCA1, which is localized in small vacuoles and not in the large central vacuole, was lacking, salt-induced (250 mM NaCl) [Ca^2+^]_cyt_ transients were more than two-fold enhanced and dramatically prolonged. Moreover, knockout plants displayed reduced expression of stress-induced genes and decreased salt tolerance [170]. Similarly, in tobacco (*Nicotiana benthamiana*) lacking the ER localized Ca^2+^-ATPase NbCA1, elicitor-induced (cryptogein) [Ca^2+^]_cyt_ transients were enhanced and programmed cell death was accelerated [171]. These studies indicate that organellar Ca^2+^-ATPases are shaping stimulus-induced [Ca^2+^]_cyt_ transients and thereby may play a role in Ca^2+^-mediated signaling.

However, when interpreting the phenotype of knockout mutants lacking specific Ca^2+^ pumps, it will always be difficult to differentiate what is a result of altered [Ca^2+^]_cyt_ transients and what is a result of changes in ion homeostasis [15]. Subtle changes in [Ca^2+^] in a certain compartment may have unexpected results. Moreover, since Ca^2+^-ATPases transport both, Ca^2+^ and H^+^, pH homeostasis might be affected as well, as shown for Ca^2+^:H^+^ exchanger of the CAX family [172].

### 3.2. Ca^2+^:H^+^ Exchanger (CAX)

Ca^2+^:H^+^ exchanger function as low affinity high turnover vacuolar Ca^2+^ pumps, driven by the electrochemical potential gradient of H^+^ (Figure 1). Flowering plants (angiosperms) have been reported to contain five to fourteen Ca^2+^:H^+^ exchanger genes. Ca^2+^:H^+^ exchanger of the CAX family are part of the Ca^2+^:cation antiporter (CaCA) superfamily, and have been detected in most eukaryotic clades; they were lost in some Mamiellales and animals (Figure 2). Plant CAXs are not selective for Ca^2+^, but transport other divalent cations, such as Mn^2+^ or Cd^2+^, as well [173,174].

Most work on Ca^2+^:H^+^ exchangers of the CAX family has concentrated on ion homeostasis, and single and double knockout mutants show varying defects not only in Ca^2+^ homeostasis but also changes in compartmentalization of other divalent cations, such as Mg^2+^ and Mn^2+^ [175,176]. Resulting phenotypes can be rather complex. An *A*. *thaliana* double knockout lacking both AtCAX1 and AtCAX3 had three-fold higher apoplastic [Ca^2+^], resulting in reduced cell wall extensibility, transpiration, and leaf growth [177]. In another study with the same double knockout (*Atcax1* & *Atcax3*) higher apoplastic pH was detected resulting in a defect in auxin transport [178]. Overexpression of *GhCAX3*, a Ca^2+^:H^+^ exchanger from cotton (*Gossypium hirsutum*), in tobacco (*N*. *benthamiana*) reduced ABA sensitivity of transgenic seedlings and affected cold stress responses, which was interpreted as an indication for a regulatory role of GhCAX3 in ABA and cold signaling [179]. Yet no [Ca^2+^]_cyt_ transients were recorded. *A*. *thaliana* knockout lines lacking either AtCAX1 or AtCAX3 display an increased ABA sensitivity of seed germination [180], and these knockout lines are known to have altered ion homeostasis [175,176]. For *CAX*, gain of function or loss of function mutants direct experimental evidence for changes in stimulus-induced [Ca^2+^]_cyt_ transients are still awaited.

## 4. Conclusions

Most work on the involvement of the vacuole in Ca^2+^ signaling in plant cells (Figure 1) has concentrated on vacuolar Ca^2+^ channels. Available data provide evidence for InsP_3_ receptor and ryanodine receptor Ca^2+^ channels, the voltage-dependent SV/TPC1 channel, and most recently for vacuolar cyclic nucleotide-gated cation channels. The phylogenetic distribution of these Ca^2+^ channels among photosynthetic eukaryotes is patchy (Figure 2) indicating a complex evolutionary history. Since no gene for InsP_3_ or ryanodine receptors has been identified in land plants, after a very promising start in the 1990s, little progress has been made in our understanding of second messenger-mediated vacuolar Ca^2+^ release. As *TPC1* was identified as encoding the SV channel, research on this field has seen a renaissance, and the SV/TPC1 channel is probably the best described ion channel in plant cells. However, we still do not understand the *in vivo* function of this channel; it is probably not Ca^2+^-induced vacuolar Ca^2+^ release. Progress in identification of genes encoding vacuolar Ca^2+^ pumps resulted in a better understanding of their physiological functions. In addition to maintaining Ca^2+^ gradients and ion homeostasis, P-type Ca^2+^-ATPases have been shown to affect the shape of [Ca^2+^]_cyt_ transients, indicating that they might contribute more to Ca^2+^ signaling then just providing the driving force for vacuolar Ca^2+^ release.
